# Characteristics of the Colombian armed conflict and the mental health of civilians living in active conflict zones

**DOI:** 10.1186/1752-1505-6-10

**Published:** 2012-11-21

**Authors:** Vaughan Bell, Fernanda Méndez, Carmen Martínez, Pedro Pablo Palma, Marc Bosch

**Affiliations:** 1 Médicos sin Fronteras España, Misión Colombia, Bogotá, Colombia; 2 Médicos sin Fronteras España, Barcelona, Spain

**Keywords:** Armed conflict, Colombia, War, Trauma, Mental health, Violence

## Abstract

**Background:**

Despite the fact that the Colombian armed conflict has continued for almost five decades there is still very little information on how it affects the mental health of civilians. Although it is well established in post-conflict populations that experience of organised violence has a negative impact on mental health, little research has been done on those living in active conflict zones. Médecins Sans Frontières provides mental health services in areas of active conflict in Colombia and using data from these services we aimed to establish which characteristics of the conflict are most associated with specific symptoms of mental ill health.

**Methods:**

An analysis of clinical data from patients (N = 6,353), 16 years and over, from 2010–2011, who consulted in the Colombian departments (equivalent to states) of Nariño, Cauca, Putumayo and Caquetá. Risk factors were grouped using a hierarchical cluster analysis and the clusters were included with demographic information as predictors in logistic regressions to discern which risk factor clusters best predicted specific symptoms.

**Results:**

Three clear risk factor clusters emerged which were interpreted as ‘direct conflict related violence’, ‘personal violence not directly conflict-related’ and ‘general hardship’. The regression analyses indicated that conflict related violence was more highly related to anxiety-related psychopathology than other risk factor groupings while non-conflict violence was more related to aggression and substance abuse, which was more common in males. Depression and suicide risk were represented equally across risk factor clusters.

**Conclusions:**

As the largest study of its kind in Colombia it demonstrates a clear impact of the conflict on mental health. Among those who consulted with mental health professionals, specific conflict characteristics could predict symptom profiles. However, some of the highest risk outcomes, like depression, suicide risk and aggression, were more related to factors indirectly related to the conflict. This suggests a need to focus on the systemic affects of armed conflict and not solely on direct exposure to fighting.

## Background

Although it is now firmly established that armed conflict has a detrimental effect on the mental health of those living in active conflict zones [1-3] it remains the case that we still know remarkably little about how different characteristics of conflict lead to specific forms of psychopathology and psychological impairment in civilians. This is likely due to the fact that great majority of the research on the psychological effects of armed conflict has been conducted on war veterans despite the fact that war leads to a greater burden on the civilian population than on soldiers [4].

Although early studies on civilians tended to focus on the impact of war on the risk of developing posttraumatic stress disorder (PTSD), it has now become clear that the effects of conflict extend beyond the direct effects of violence to include a host of social and economic hardships that can be as equally important in determining the likelihood of developing a mental illness [5]. Furthermore, epidemiological work has shown that PTSD is only one of a number of possible outcomes after catastrophic and violent events with trauma raising the risk of a wide range of psychiatric disorders [6].

Perhaps unsurprisingly, the evidence to date suggests that armed conflict has a powerful negative effect on the mental health of civilians. The majority of this evidence comes from retrospective studies that report a clear association between mass violence and poor long-term psychological outcomes in adults civilians from Afghanistan [7,8], Lebanon [9], Rwanda [10], the Balkans [4], Cambodia [11,12], Ethiopia and Algeria [1,13] among others [14]. However, aside from this research being retrospective (and, therefore, subject to the relevant biases of recall based data) it remains the case that these studies provide evidence related to mental health in post-conflict situations whereas there is still an urgent need to understand the mental health of civilians in ongoing conflict.

Studies that address the impact of ongoing conflict are limited in their number compared to post-conflict research, although the research that does exist indicates that mental health remains a significant issue. Research on the Israeli-Palestinian conflict has found that both Palestinian and Israeli populations have high levels of psychiatric morbidity associated with the ongoing conflict with social support being a significant mediator of outcome (e.g. [15,16]). Similarly, the 2006–7 Iraq mental health survey conducted by the World Health Organisation [17] during a period of intense nationwide sectarian violence reported exposure to bomb blasts, mutilated bodies and gunfire were associated with an increased risk of mental disorder. While not specifically on people who were residing in active war zones, research conducted in Nepal on internally displaced persons still at risk from violence indicated high rates of posttraumatic symptoms [18] with victims of torture likely to present with PTSD, persistent somatoform pain disorder, affective disorders, generalized anxiety disorder, and dissociative symptoms [14,19].

Although research on ongoing conflict is limited, it is particularly striking that there is very little research from Latin America, which, as a region, has a long history of armed conflict from the 20^th^ Century which, in some cases, has continued into the 21^st^. Particularly notable is the case of Colombia which has seen armed conflict since 1964 (a situation still ongoing at the time of writing) that has had an extensive impact on the civilian population with widespread reports of human rights abuses [20]. Consequently, Colombia has one of the highest number of internally displaced people due to violence in the world, estimated at between 3.3 and 4.9 million people [21].

Despite the fact that the profile of the conflict would suggest a heavy toll on the mental health of the population, surprisingly little systematic work has aimed to characterise and quantify the impact on the population. Sanchez-Padilla et al. [22] reported high levels of psychopathology in patients attending a mental health clinic in the conflict-affected state of Tolima although no analysis was conducted to examine the link between specific conflict-related events and psychopathological outcomes. In people displaced by the armed conflict to a non-affected city, Cáceres et al. [23] reported that 80% of the study participants had experience violence related to the conflict (although no mental health outcomes were reported) while almost 30% of displaced people living in an urban slum were found to have a common mental disorder by Puertas et al. [24]. High levels of PTSD, often with similarly high levels of comorbid anxiety and depression, have been reported in adults displaced by the armed conflict in three studies [25-27] with similar findings reported in children [28], although the conclusions are drawn from studies with relatively small sample sizes. Still lacking, however, is research on the mental health of civilians resident amid the areas of active conflict in Colombia. This could provide evidence not only for national mental health services but also could help elucidate the impact of ongoing mass violence on civilians in general.

As an independently funded neutral and impartial organisation *Médecins Sans Frontières* (MSF) provides mental health services in several of the Colombian conflict zones, many of which are not served by state services due to economic, geographical or security reasons. This study uses clinical data from these services to provide epidemiological information from patients consulting in the active conflict zones of Colombia while testing the prediction that more severe exposure to conflict violence would be associated with more serious psychopathology.

## Method

### Procedure

*Médecins Sans Frontières* works in the south of Colombia in the rural departments (departments are equivalent to states) of Caquetá, Cauca, Valle de Cauca, Nariño and Putumayo where the organisation provides consultations with medical professionals and psychologists in fixed health centres or mobile clinics (mobile clinics enter areas for a limited time - for example, three days a month). Patients are attracted to the clinic by posters, leaflets and promotion in the local community, including talks with community groups that outline common health issues and how they can be managed. The areas of intervention are chosen due to them being affected by the ongoing conflict in Colombia and due to a lack of primary and mental health services.

When a patient consults with a psychologist he or she is assessed and a clinical history form is completed which contains both a narrative account of the patient and the intervention as well as standardised sections for recording demographics, risk factors and symptoms. The standardised data is later entered into an anonymised database which forms the basis of this study. Patients are coded by serial number with their sex, age, source of referral, date of consultation, location of consultation, symptoms and risk factors. Symptoms and risk factors are from a standardised list and clinicians are asked to select up to three symptoms and up to four risk factors which are coded in a yes / no (present / absent) fashion to best capture the presenting clinical issue. The list of symptoms and risk factors were developed and revised over the lifespan of the project to be of pragmatic use to the clinicians on the ground and as an aid for clinical service audits, while the limitation on recording a certain number of items was due to the limitations in the clinical records software although this number was originally chosen based on the number of items recorded in typical sessions.

The data included in this study is from the period of January 2010 until the end of November 2011 during which 8,100 individual patients attended the clinics. The data is taken from the evaluation completed on the initial visit and the selection criteria for this study included patients 16 years or older, leaving 6,353 patients in the analysis. As this study involved the retrospective analysis of anonymised data collected as part of routine clinical operations, consistent with the Declaration of Helsinki (Principal 1), no formal ethical review was required. However, a proposal for the study was submitted to the organisation’s Research Committee, who evaluated and approved the study before the analysis was begun.

### Analysis

Owing to the large number of risk factors and owing to a desire to uncover links between the general underling characteristics of the conflict and individual symptoms, all risk factors were entered into a hierarchical cluster analysis. Hierarchical cluster analysis groups items into clusters based on their statistical co-occurrence and these clusters are interpreted thematically post-hoc by the researchers. The analysis of variables for the hierarchical cluster analysis was completed using IBM SPSS version 19.0 using Ward’s method with squared Euclidean distance similarity. Hierarchical binary logistic regression analyses were then used to test the predictive value of each risk factor cluster on the presence of individual symptoms. To control for the effect of demographic variables, variables were entered in two blocks with sex and age in the first block and risk factor clusters in the second, with demographic-adjusted associations between symptoms and risk factor clusters reported here. The associations were summarised as the estimated odds ratios with 95% confidence intervals.

## Results

Of the 6,353 patients, 1,540 (24.2%) were male and 4,814 (75.8%) were female. There was a significant difference between the mean age of males (39.5 years old; SD = 15.4) and females (35.78 years old; SD = 13.7) with males tending to be slightly older (t_(6352)_ = −8.893; *p* < .0005). Prevalence of the symptoms and risk factors are displayed in Tables 1 and 2.

**Table 1:** Prevalence of symptoms in sample

***Symptom***	***N***	%
Low mood	2974	46.8
Excessive worry and hopelessness	2534	39.9
Fear, feeling of threat	1488	23.4
Anxiety symptoms	1265	19.9
Weeping	1060	16.7
Sleep disorders	1054	16.6
Generalised somatic body pain	889	14.0
Aggression	751	11.8
Intrusive thoughts / feelings	629	9.9
Guilt / self hate	488	7.7
Emotional numbing	460	7.2
Weakness, fatigue, lack of energy	385	6.1
Avoidance	350	5.5
Reproductive and sexual problems	324	5.1
Suicidal ideation / attempts	275	4.3
Hyperactivity / lack of concentration	213	3.4
Eating / feeding disorders	191	3.0
Alcohol / substance abuse	144	2.3
Magical and religious ideation	115	1.8
Psychotic symptoms	86	1.4
Enuresis / encopresis	18	0.3
Menstrual problems	8	0.1
No symptoms recorded	24	0.4

**Table 2:** Prevalence of risk factors in sample

***Risk factor***	***N***	%
Problems in family social support network	3059	48.1
Living alongside members of armed groups	2530	39.8
Economic problems	1849	29.1
Problems in wider social support network	1374	21.6
Exposure to other violence (physical or psychological)	1004	15.8
Female abuse	1004	15.8
Forced displacement	952	15.0
Problems in social support network related to conflict	827	13.0
Violent death of significant person	655	10.3
Abandonment and negligence (object)	558	8.8
Death of significant person	560	8.8
Direct threat from armed group	549	8.6
Exposure to explosives	532	8.4
Witness to sexual violence against significant person	26	4.0
Victim of sexual violence	235	3.7
Abandonment and negligence (subject)	230	3.6
Unwanted pregnancy	2001	3.1
Child abuse	179	2.8
Disappearance of significant person	117	1.8
Detention or kidnapping of significant person	103	1.6
Patient is a member of an armed group	104	1.6
Displacement due to fumigation^1^	71	1.1
Witness to torture of significant person	69	1.1
Forced recruitment of significant person	55	0.9
No risk factors recorded	45	0.7

^1^ Aerial fumigation is used in Colombia to eradicate coca crops as part of government anti-cocaine trafficking measures. Those who rely on coca for their livelihood or those who have their food crops destroyed by the same process may be forced to leave their dwellings.

The analysis produced three main clusters, clearly showing a cluster of risk factors groups as ‘violence directly related to the conflict’, a cluster grouped as ‘personal violence not directly related to conflict’ and a cluster groups as ‘general hardship’ which do not contain risk factors describing experience of personal violence.

The labeled clusters and their components are shown below:

Cluster 1. Violence directly related to the conflict

Disappearance of significant person

Retention or kidnapping of significant person

Direct threat from armed group

Exposure to explosives

Living alongside members of armed groups

Forced recruitment of significant person

Witness to torture of significant person

Patient is a member of an armed group

Problems in social support network directly related to the conflict

Death of significant person

Violent death of significant person

Forced displacement

Cluster 2. Personal violence not directly related to conflict

Female abuse

Child abuse

Abandonment and negligence (object)

Victim of sexual violence

Exposure to other violence (physical or psychological)

Cluster 3. General hardship

Abandonment and negligence (subject)

Witness to sexual violence against significant person

Problems in family social support network

Problems in wider social support network

Displacement due to fumigation

Economic problems

Unwanted pregnancy

For the binary logistic regression analyses the symptoms ‘enuresis / encopresis’ and ‘menstrual problems’ were rejected apriori due to low prevalence rates (N = 18 / 0.3% prevalence and N = 8 / 0.1% prevalence respectively). The models contained five independent variables which were entered in two blocks. To derive the predictive ability of the risk factor clusters after demographic variables had been accounted for the variables were entered in two blocks: age and sex were entered in the first block while risk factor clusters were entered in the second block. All full models were significant at *p* < 0.005 except the model for ‘psychotic symptoms’ which was rejected. Furthermore, results for the symptoms ‘excessive worry and hopelessness’, ‘avoidance’, ‘reproductive and sexual problems’ and ‘guilt, self hate’ were rejected due to poor model fitting as reflected in a significant Hosmer and Lemeshow test result. Results from the remaining logistic regressions are show in Table 3 (sex and age) and Table 4 (recorded risk factors). The odds ratios describe the odds of a symptom being present for every additional unit of the independent variables as follows: age per increasing year and sex with male sex, and for every additional risk factor present in the clusters ‘violence directly related to the conflict’, ‘personal violence not directly related to conflict’ and ‘general hardship’. In other words, for the odds ratios related to age, values greater than one show the symptom is more common in older age, values less than one with younger age. For odds ratios related to sex, values greater than one indicate that the symptom is more common in males, values less than one that the symptom is more common in females. For risk factor clusters, odds ratios greater than one indicate that the symptom is more common as the number of risk factors in the cluster increases, and odds ratios less than one indicate that the symptom is less common as the number of risk factor related to the cluster increases.

**Table 3:** **Results of association between block one variables** (**sex and age**) **and recorded symptoms**

	***Age***			***Sex***		
	***p***	***OR***	***95***% ***CI***	***p***	***OR***	***95***% ***CI***
Aggression	<0.0005	0.98	0.97-0.99	<0.0005	1.71	1.41-2.05
Alcohol / substance abuse	0.356	1.00	0.98-1.01	<0.0005	6.44	4.49-9.22
Anxiety symptoms	0.081	1.00	0.99-1.00	0.073	1.14	0.99-1.32
Suicidal ideation / attempts	<0.0005	0.98	0.97-0.99	<0.0005	1.69	1.28-2.24
Low mood	0.451	1.00	0.99-1.01	<0.0005	0.61	0.54-0.68
Eating / feeding disorders	0.058	1.01	1.00-1.02	0.004	0.54	0.36-0.82
Fear, feeling of threat	0.097	1.00	0.99-1.00	0.725	0.97	0.84-1.13
Generalised somatic body pain	<0.0005	1.03	1.02-1.03	0.540	1.05	1.11-1.29
Hyperactivity / lack of concentration	0.003	0.98	0.97-1.00	0.175	0.81	0.60-1.01
Intrusive thoughts / feelings	0.126	1.00	0.99-1.00	<0.0005	0.70	0.58-0.85
Sleep disorders	<0.0005	1.03	1.02-1.03	<0.0005	0.62	0.54-0.72
Weakness, fatigue, lack of energy	0.037	1.01	1.00-1.02	0.897	1.02	0.80-1.29
Magical and religious ideation	0.016	1.02	1.00-1.03	0.037	0.65	0.44-0.98
Weeping	0.032	1.01	1.00-1.01	<0.0005	2.81	2.30-3.43
Emotional numbing	<0.0005	0.98	0.98-0.99	0.170	1.18	0.93-1.49

p = statistical significance; OR = standardised odds ratio (standardised beta); CI = confidence intervals.

**Table 4:** **Results of association between block two variables** (**risk factor clusters**) **and recorded symptoms**

	***Violence directly related to the conflict***			***Personal violence not directly related to conflict***			***General hardship***		
	***p***	***OR***	***95%CI***	***p***	***OR***	***95%CI***	***p***	***OR***	***95%CI***
Aggression	0.285	0.95	0.87-1.04	<0.0005	1.78	1.60-1.97	<0.0005	1.22	1.12-1.39
Alcohol / substance abuse	0.984	1.00	0.83-1.22	<0.0005	1.73	1.40-2.20	0.25	1.31	1.03-1.66
Anxiety symptoms	<0.0005	1.13	1.06-1.21	0.014	0.89	0.80-0.98	0.75	1.01	0.93-1.11
Suicidal ideation / attempts	<0.0005	1.42	1.23-1.65	<0.0005	2.32	1.96-2.74	<0.0005	1.73	1.45-2.07
Low mood	<0.0005	1.33	1.26-1.40	<0.0005	1.50	1.38-1.61	<0.0005	1.24	1.15-1.33
Eating / feeding disorders	0.017	1.21	1.04-1.42	<0.0005	1.51	1.24-1.85	0.776	1.03	0.83-1.28
Fear, feeling of threat	<0.0005	1.76	1.65-1.88	<0.0005	1.22	1.12-1.34	0.001	0.86	0.78-0.94
Generalised somatic body pain	<0.005	1.19	1.10-1.29	0.767	1.02	0.91-1.14	0.020	1.13	1.02-1.25
Hyperactivity / lack of concentration	0.163	0.90	0.77-1.04	0.001	0.65	0.51-0.83	0.131	1.15	0.96-1.34
Intrusive thoughts / feelings	0.014	1.12	1.02-1.22	0.020	1.16	1.02-1.31	0.856	0.99	0.88-1.12
Sleep disorders	<0.0005	1.26	1.17-1.35	0.365	1.05	0.94-1.17	0.012	1.13	1.03-1.25
Weakness, fatigue, lack of energy	0.145	0.92	0.82-1.03	0.003	0.76	0.64-0.91	0.124	1.12	0.97-1.29
Magical and religious ideation	0.188	1.15	0.93-1.41	0.138	1.24	0.93-1.66	0.003	1.47	1.14-1.90
Weeping	<0.0005	1.36	1.27-1.46	<0.0005	1.31	1.19-1.44	<0.0005	1.25	1.14-1.38
Emotional numbing	0.408	0.95	0.85-1.07	0.552	0.96	0.82-1.11	<0.0005	1.63	1.43-1.85

p = statistical significance; OR = standardised odds ratio (standardised beta). CI = confidence intervals.

Owing to the high number of comparisons, a Bonferroni correction was applied and symptoms significantly associated with each predictor were selected on the basis of having a *p* value less than 0.001. As can be seen from the results, age is generally not associated with symptoms and when the association is significant the effect size is small. In contrast, gender is strongly and significantly associated with several symptoms, with males more likely to experience substance abuse, weeping, aggression, suicidal thoughts and actions and female more likely to experience intrusive thoughts and feelings and sleep disorders. With regard to the three risk factor clusters, conflict related violence has a greater association with anxiety related symptoms (e.g. fear, feeling of threat: OR = 1.76; sleep disorders or difficulties: OR = 1.26; anxiety symptoms: OR = 1.13), while non-conflict related violence was associated with a higher prevalence of impulsivity symptoms such as suicidal ideation and attempts (OR = 2.32), aggression (OR = 1.78) and substance abuse (OR = 1.73). The general hardship cluster had a broader symptom association including suicidal ideation and attempts (OR = 1.73), emotional numbing (OR = 1.63) and magical ideation (OR = 1.47). Notably, depressive symptoms seem equally represented across all three clusters. For example, in all three clusters there is an association with low mood (OR range 1.24 – 1.50), weeping (OR range 1.25 – 1.36) and suicidal ideation and attempts (OR range 1.42 – 2.32).

## Discussion

This analysis of data from civilians consulting with mental health services in active conflict zones of Colombia indicated that recorded risk factors fall into three groups labelled ‘violence directly related to the conflict’, ‘personal violence not directly related to conflict’ and ‘general hardship’. The regression analyses indicated that depression-related symptoms and suicide risk symptoms (e.g. ‘Low mood’ ‘Suicidal ideation / attempts’) were frequent across all clusters, however, anxiety-related symptoms (e.g. ‘Fear, feeling of threat’ and ‘Anxiety symptoms’) were more commonly related to ‘violence directly related to the conflict’ while impulsivity-related symptoms (e.g. ‘Aggression’ and ‘Alcohol / substance abuse’) were more related to ‘personal violence not directly related to conflict’.

The results of this study provide additional evidence that non-conflict related factors are equally as important in determining mental health outcome in people affected by mass violence [5]. In contrast to the study’s prediction, suicidal ideation and attempts were as strongly associated with non-conflict violence and general hardship as violence directly related to the conflict, suggesting that this could be a general effect associated with living in areas of mass violence and not something clearly associated with specific characteristics of the context. Nevertheless, as the single most strongly associated mental health outcome across risk factor clusters it does highlight a clear risk for suicide in conflict affected civilian populations. As Colombia has a one of the highest rates of suicide risk behaviour in the world [29] and suicide rate has been shown to rise in post-conflict populations [30] this may continue to be a significant public health issue in the future.

While low mood and depression-related symptoms seem to be equally represented across the clusters it is notable that ‘violence directly related to the conflict’ was more commonly associated with anxiety and arousal-related symptoms (‘Fear, feeling of threat’, ‘Sleep disorders or difficulties’ and ‘Anxiety symptoms’) than the ‘personal violence not directly related to the conflict’ cluster which contains a larger number of associations with impulsivity-related symptoms (‘Suicidal ideation / attempts’, ‘Aggression’ and ‘Alcohol / substance abuse’). Owing to the cross-sectional nature of the study, it is not possible to deduce causality from the data and it would be difficult to predict apriori whether people who are impulsive are more likely to experience non-conflict related violence or vice versa. However, it is perhaps more likely that ‘violence directly related to the conflict’ increases the risk for anxiety-related symptoms rather than the other way round, owing to its conceptual coherence and substantial evidence that conflict-related violence increases risk for anxiety disorders [14].

In contrast to other research showing that older people had greater levels of psychopathology, age had a minimal effect on mental health outcomes. While several symptoms were significantly associated with age, odds ratios ranged from 0.98 to 1.03 indicating a negligible effect size. Nevertheless, there was a large effect of sex on several symptoms with ‘alcohol / substance abuse’ being significantly more likely to be present in males. This association is in line with previous studies where alcohol and substance use is more common in civilian males after experience of armed conflict [31,32]. As also might be expected, aggression was significantly more associated with males in the study, although perhaps less expected would be the association with weeping. However, it needs to be remembered that as a clinical study the data represents not only the effect of armed conflict on the patients but also what causes the patients to consult with the mental health service. It is possible that weeping may act to motivate male patients or others around them to request a consultation due to the fact that it is culturally less acceptable for men to weep potentially signalling a ‘need for help’.

In interpreting the data from this study it is important to bear in mind its limitations. There is a chance of selection bias in that the study included data only from those that consulted with MSF mental health clinics. It is possible that those who were most disabled were less likely to attend or that cultural factors may have influenced which problems were most likely to present in the clinics. Similarly, lack of resources or geographical obstacles may impede movement in rural areas of Colombia, as can the conflict itself in that some populations can be prevented from moving through or leaving certain zones by armed groups. The data does not distinguish between acute and chronic stressors and it is possible that the impact of different risk factors depends on their duration and time course. Importantly, the fact that only up to three symptoms and up to four risk factors can be recorded may have reduced the strength of associations between variables. Similarly, the fact that data were drawn from clinical assessments rather than standardised measures may mean that a wider range of conflict characteristics and psychopathology may be related which are not fully captured by the clinically-oriented assessments.

## Conclusions

As one of the few studies on the mental health of civilians in active conflict zones and some of the only evidence of the impact of the Colombian armed conflict on civilians this study provides evidence of a significant burden for those caught up in hostilities. Nevertheless, it is clear from the findings that mental health burden is not solely related to direct experience of armed violence as some of the most serious outcomes, like suicide-risk, depression and aggression, were linked equally or more strongly to experiences not directly associated with the conflict. It is important to understand armed conflict as having a systemic effect on the risk for mental illness, which, while also including direct experience of conflict-related violence, will also include disruption to social support networks, increased anti-social behaviour, poverty, a limited ability to access essential services and range of other interconnected effects. It is therefore important that interventions and treatment programmes for the affected populations are not solely trauma focussed but include a range of social and clinical aspects to address the diversity of social problems and mental heath outcomes.

## Abbreviations

PTSD: Posttraumatic stress disorder; MSF: Médecins Sans Frontières; SD: Standard deviation; OR: Odds ratio; CI: Confidence interval.

## Competing interests

The authors declare that they have no competing interests.

## Authors’ contributions

VB, FM, CM, PPP and MB were involved with the design of the study and writing of the manuscript. VB conduced the analysis and interpretation of the data. All authors read and approved the final manuscript.
